# Occupational Performance and Quality of Life in Individuals With Cardiovascular Conditions: A Cross‐Sectional Study

**DOI:** 10.1155/oti/4141955

**Published:** 2026-03-30

**Authors:** Ala’a Jaber, Raja’a Alzoubi, Sukainah Rawashdeh, Ala’a Oteir, Mohammad Nazzal, Saad Al-Naasan, Noor Ismael

**Affiliations:** ^1^ Department of Rehabilitation Sciences, Jordan University of Science and Technology, Irbid, Jordan, just.edu.jo; ^2^ Department of Internal Medicine, King Abdullah University Hospital, Irbid, Jordan, kauh.jo; ^3^ Department of Allied Medical Sciences, Jordan University of Science and Technology, Irbid, Jordan, just.edu.jo; ^4^ Department of Physical and Occupational Therapy, Hashemite University, Zarqa, Jordan, hu.edu.jo

**Keywords:** activities of daily living, cardiovascular disease, occupational performance, quality of life

## Abstract

**Introduction:**

Cardiovascular conditions significantly impact individuals’ functional abilities. Existing evidence is limited on levels of occupational performance in individuals with cardiovascular conditions and what daily life activities are impacted. This study aimed to investigate occupational performance and quality of life in this population.

**Methods:**

This current study utilized a cross‐sectional design and convenient sampling. The sample consisted of 52 individuals with cardiovascular conditions. Instruments included Demographic and Health Condition Questionnaire, The Canadian Occupational Performance Measure (COPM), and The World Health Organization Quality of Life‐BREF (WHOQOL‐BREF). Data analyses included descriptive statistics, analysis of frequencies, Spearman rank‐order correlations, and exploratory multivariable linear regression analyses.

**Findings:**

Individuals with cardiovascular conditions perceived their occupational performance and satisfaction in the lower moderate range (COPM Performance: mean = 4.74, SD±1.75, range 1–8; COPM Satisfaction: mean = 4.5, SD±2.04, range 1–10). Self‐care was the most common domain with occupational performance challenges (66.2% of all reported problems). Functional mobility was the most frequently reported self‐care problem. In the WHOQOL‐BREF, the physical domain was the lowest (M = 50.17, SD ±18.4, Range = 0–88). Significant predictor of better occupational performance included having higher physical health‐related QoL (*R* ^2^ = 0.158, *p* = 0.005). Significant predictors of greater satisfaction with occupational performance included participation in sports and having a better psychological QoL (*R* ^2^ = 0.232, *p* = 0.048).

**Conclusion:**

Occupational performance in daily life activities in individuals with cardiovascular conditions, and their quality of life are negatively impacted. Individualized interventions for this population should target daily life activities, especially self‐care.

**Key Findings**

• Individuals with cardiovascular conditions have low to moderate occupational performance and satisfaction in self‐care, productivity, and leisure activities.
• Functional mobility, sleep, and heavy work are the most challenging daily life activities.
• Low occupational performance and satisfaction are related to low physical, psychological, and environmental quality of life in this population.
• Predictors of high occupational performance and satisfaction included engaging in sports activities, higher physical health‐related quality of life, and a stable psychological aspect of quality of life.

## 1. Introduction

Occupational performance in daily life activities is challenged by several health conditions, especially those affecting the cardiovascular system. It is evident in the literature that people’s ability to perform different daily activities like self‐care or work affects their health, and quality of life, e.g., [1, 2]. Considering how cardiovascular conditions seriously affect people’s lives, these conditions affect people’s ability to continue their life roles, functions, and participation [3, 4]. Limited evidence looked at occupational performance of individuals with cardiovascular conditions [1]. Up to authors’ knowledge, there are no studies that investigated how occupational performance in this population affects quality of life. Therefore, there is a need to build on the body of evidence about what activities are most challenging in individuals with cardiovascular conditions, and how levels of occupational performance in these activities affect quality of life.

## 2. Literature Review

Cardiovascular conditions affect the heart and its blood vessels. These conditions are classified into four entities: coronary artery disease (CAD), cerebrovascular disease, peripheral artery disease (PAD), and aortic atherosclerosis [5]. Heart failure, in which the heart does not pump blood properly around the body [6], is a consequence of CAD that results from decreased blood flow to the heart muscle [5, 6]. Cardiovascular conditions are the leading cause of mortality globally [7]. The prevalence of cardiovascular conditions is increasing worldwide due to many reasons including the change in daily life activities from physically demanding ones to sedentary, and technology‐dependent lifestyle [8], adding to that the high fat diet consumption with less vegetables and fruits, and increased obesity rates. While there are many risk factors for cardiovascular conditions, people’s daily life activities and routines (i.e., smoking, unhealthy diet) play a tremendous role in increasing the risk of having these conditions [9]. Not changing the way individuals with cardiovascular conditions perform their daily activities and routines will affect their health and quality of lives, and may lead to serious complications and death [10].

The Canadian Model of Occupational Performance and Engagement (CMOP‐E) [11] indicates that personal and health characteristics (e.g., physical abilities), daily life activities and routines (e.g., job demands), and supporting environment (e.g., physical workspace) can significantly affect occupational performance in daily life activities including self‐care, productivity, and leisure. Occupational performance is referred to as the way people perform their daily life activities in its authentic contexts. People evaluate the process of performing activities and also the end product of these activities based on their values, beliefs, and cultural expectations. In applying this theoretical framework to individuals with cardiovascular conditions, this theory suggests that the physical and health characteristics in this population, their activities and routines, and contexts of their activities affect the way they perform daily life occupations. Therefore, investigating occupational performance in individuals with cardiovascular conditions should account for these factors.

Cardiovascular conditions adversely influence the physical [12], cognitive [13], and psychosocial abilities [14], leading to functional limitations and disability [15]. Existing studies on occupational performance for individuals with cardiovascular conditions showed that these individuals experience challenges in performing everyday activities due to physical abilities’ decline, and failure to make adaptations to everyday activities [16]. A study investigated limitations in daily activities in individuals with cardiovascular conditions and reported that walking, climbing up the stairs, bathing, dressing, and outings were the most challenging ones [17]. A qualitative study of occupational performance in elderly with chronic heart failure uncovered an important theme that described how those individuals realized their limited activity ability, their challenge to preserve an active life while focusing on meaningful activities, and their struggle in changing habits and roles [18].

Evidence investigating occupational performance in individuals with cardiovascular conditions is limited in number of studies and methods used to assess occupational performance, e.g., [16–18]. Existing studies on occupational performance included mostly individuals with heart failure, that is, [18], in which occupational performance in other mild cardiovascular conditions remains unclear. Also, many existing studies focused on the pediatric population in which occupational performance areas are different than adults and older adults [19]. Existing studies did not utilize a comprehensive occupational performance assessment tool that specifically showed what daily life activities were impacted. For example, a study used a non‐standardized qualitative questionnaire to identify occupational performance limitations among 10 individuals with chronic heart failure, and hence many daily life areas remained under investigated [18]. Adding to this, there is a need to update and build the body of evidence about occupational performance in this population especially that there are no studies in this field in Jordan or the Middle East. A relatively recent systematic review and meta‐analysis has showed that the Middle East had an overall cardiovascular conditions’ prevalence of 10% of its population [20]. Also, the middle east, especially Jordan, has a very high prevalence of cardiovascular risk factors including smoking and hypertension [21]. The unique and individualized occupational therapy and rehabilitation for individuals with cardiovascular condition require additional research that guides tailored evaluation and intervention. Therefore, this study aimed to investigate occupational performance challenges in a variety of daily life activities in individuals with cardiovascular conditions. In addition, this study explored relationships between occupational performance, quality of life domains, and other demographic factors.

## 3. Method

### 3.1. Study Design

The current study utilized a cross‐sectional design and convenient sampling to explore occupational performance challenges and quality of life aspects among individuals with cardiovascular conditions.

### 3.2. Participants and Settings

The inclusion criteria consisted of the following: (1) adults aged 18 years or older, (2) who had a diagnosis of a cardiovascular condition from their cardiologists, (3) had their diagnosis for at least 3 months before participating in this study in order to be considered as a stable condition (as recommended by a cardiologist, third author), (4) were receiving health services in outpatient clinics and were not in‐patients at the time of the study, and (5) were able to read and write in Arabic in order to complete the study tools. The study excluded the following: (1) Individuals below 18 years, (2) who were admitted to a hospital at the time of the study with an acute cardiovascular condition, (3) had the condition for a period less than 3 months, (4) had other health conditions that can impact occupational performance and quality of life (e.g., neurological or psychosocial conditions), and (5) were not able to complete the study tools because of communication challenges.

### 3.3. Outcome Measures

Demographic and Health Condition Questionnaire. Researchers in the current study designed this tool to collect personal (e.g., age, weight, gender, marital status, employment status) and health‐related information (e.g., smoking, cardiac disease diagnosis, comorbid conditions, and past medical history).

The Canadian Occupational Performance Measure [22]. The COPM evaluates self‐perceived occupational performance and satisfaction about performing daily life areas including self‐care, productivity or work, and play or leisure. When using this outcome measure, health‐care providers perform semi‐structured interviews with clients to determine challenges in occupational performance through a four‐steps process: (1) clients identify occupational performance in the three main COPM areas, (2) clients rate the importance of each problem using a scale from 1 (*not important at all*) to 10 (*extremely important*), (3) clients choose up to five occupational performance problems from the three main areas focusing on the importance rating performed in the previous step, and finally, (4) clients rate the top five occupational performance problems based on performance (the way they do the activity) and satisfaction (how satisfied they are with the way they do the activity) both using a scale from 1 (*not able to do it at all/not satisfied at all*) to 10 (*able to do it extremely well/extremely satisfied*).

While COPM calculations yield occupational performance total scores (5–50), mean scores (total scores/five) are mostly used by researchers and healthcare providers. This study referred to Jaber et al. to classify mean occupational performance and satisfaction (poor = 1–3, moderate = 4–6, good = 7–10) [23]. A number of psychometric studies on different populations have supported the validity and reliability of the COPM, e.g., [24–27]. The COPM semi‐structured interviews can be done face‐to‐face or via telephone [25]. COPM authors suggested that remote semi‐structured interviews are possible especially for re‐assessment after intervention [25]. A study by Carr and Worth in 2001 has shown that face‐to‐face and phone interviews yield comparable data [28].

The World Health Organization Quality of Life‐BREF. This brief 26‐item tool assesses four quality of life (QoL) domains: Physical (seven items, e.g., To what extent do you feel that physical pain prevents you from doing what you need to do?), psychological (six items, e.g., To what extent do you feel your life to be meaningful?), social relationships (three items, e.g., How satisfied are you with your personal relationships?), and environment (eight items, e.g., How healthy is your physical environment?). WHOQOL‐BREF utilizes a five‐point Likert scale (1 = *very poor or not at all or dissatisfied* to 5 = *very good or always or very satisfied*) in activities that enhance QoL, and provides domain scores on a scale from zero to a hundred. Cut‐off scores for each domain as follows: 50 for physical, 51 for psychological, 50 for social relationships, and 58 for the environment [29]. Several studies utilized the WHOQOL‐BREF with different populations, e.g., [30, 31]. Also, psychometric studies confirmed the validity and reliability of this tool, e.g., [32, 33].

### 3.4. Procedures

Researchers obtained ethical approval from the Institution’s Internal Review Board (Research #20200205). A cardiologist (third author) provided a list of potential participants with confirmed cardiovascular diagnoses. Recruitment efforts further included social media platforms (e.g., Facebook) to expand sampling and to increase participant enrollment. A researcher in this study (second author) contacted 91 potential participants by phone to determine if they were eligible for participation, and explained in details the study purposes, procedures, and potential benefits and risks. Of these, 39 potential participants were excluded for various reasons such as presence of neurological or musculoskeletal condition (*n* = 10), acute cardiac disease (*n* = 1), or refusal to participate (*n* = 28). Participants who met the eligibility criteria were asked to provide their verbal informed consent prior to enrollment. The use of verbal consent was approved by the IRB and was documented using a standard consent script with the date and time recorded for each participant. Verbal informed consents were documented by the second author using detailed note taking including the date and the time. The researcher used a standardized consent script to ensure consistency between participants. The eligible 52 participants then completed an interview containing the study’s outcome measures (demographic and health condition questionnaire, COPM open‐ended questions, and WHOQOL‐BREF questionnaire). All phone interviews were performed by one researcher to improve measurement consistency and to avoid interrater bias. The researcher used a unified script for asking questions during phone interviews with participants to reduce the risk of data collection bias. Phone interviews ranged between 45 min and 1 h depending on the number of occupational performance challenges that were determined by study participants. All questionnaires were fully collected from all 52 participants and no missing data were observed in this study.

### 3.5. Statistical Analyses

Data analyses utilized the Statistical Package for the Social Sciences [34]. Data analyses included descriptive statistics to summarize sample demographics, and COPM and WHOQOL‐BREF domain scores. An analysis of frequencies showed occupational performance challenges that were categorized by the main COPM domains (i.e., Self‐care, Productivity, Leisure) and by the Occupational Therapy Practice Framework [35] (i.e., showering and grooming as self‐care activities). The variables were tested for normal distribution using the Shapiro–Wilk test, which indicated the use of Spearman Rank‐Order correlations between COPM and WHOQOL‐BREF domain scores (Psychological domain *p* = 0.01; social relationships domain *p* = 0.03). This study referred to Dancey and Reidy to determine the strength of correlations: Strong (*r*_*s*_ = 0.7 or higher), moderate (*r*_*s*_ = 0.69–3), and weak (*r*_*s*_ = 0.29 or lower) [36]. An exploratory multivariable linear regression analysis using stepwise selection method was conducted to identify predictors of COPM performance and satisfactions scores. The independent variables entered into the predictive models included the sample’s demographic characteristics and the WHOQOL‐BREF domain scores. The assumptions of the multivariable linear regression (i.e., normality, linearity, and homoscedasticity) were assessed and confirmed to be satisfactory. Multicollinearity was assessed for highly correlated independent variables with a high variance inflation factor (VIF ≥ 5), and addressed by removing one of the two correlated independent variables from the list of potential predictors. Because of the exploratory nature of the regression analysis in this study, no adjustments were made for multiple comparisons. A *p* value threshold of 0.10 was applied for the removal of independent variables from the regression model [37]. Statistical significance for all analyses was defined as a *p* value less than 0.05.

## 4. Findings

The sample consisted of 52 individuals with chronic cardiovascular conditions, mean age 58.48 years (SD = ±12.69, range = 24–90 years), and mean duration of condition 8.3 years (SD±7.1, range = 1–40 years). Around a third of the participants (36.9%) had a combination of three other health problems related to cardiovascular conditions (i.e., hypertension, diabetes mellitus, and high cholesterol), and around half (51.9%) underwent surgical procedures, mainly catheterization, followed by coronary artery bypass grafting, and valve replacement. See Table 1 for additional demographic and health condition characteristics of the sample.

**Table 1:** Demographic and condition characteristics of the sample (*N* = 52).

**Characteristics**	**Central tendency statistics**
Age	
Mean	58.48 years
SD	±12.687
Range	24–90
Weight	
Mean	83.46 kg
SD	±17.099
Range	52–120
Duration of having cardiac problems in years	
Mean	8.31
SD	±7.086
Range	1–40

**Characteristics**	**Frequencies *n*, (%)**
Age categories	
20–29	1, (1.9%)
30–39	1, (1.9%)
40–49	9, (17.3%)
50–59	18, (34.6%)
60–69	15, (28.9%)
70–79	3, (5.8%)
80–89	4, (7.7%)
90–100	1, (1.9%)
Gender	
Male	43, (82.7)
Female	9, (17.3)
Marital status	
Single	3, (5.8)
Married	41, (78.8)
Widower	7, (13.5)
Divorced	1, (1.9)
Smoking	
Yes	18, (34.6)
No	34, (65.4)
Playing sport	
Yes	18, (34.6)
No	34, (65.4)
Employment status	
Employed	17, (32.7)
Unemployed	35, (67.3)
Cardiovascular condition	
CD	23, (44.2)
CAD	29, (55.8)
Comorbid conditions	
HTN	9, (17.3)
Diabetes	6, (11.5)
High cholesterol	2, (3.8)
HTN and high cholesterol	2, (3.8)
HTN and diabetes	5, (9.6)
Diabetes and high cholesterol	1, (1.9)
HTN and diabetes and high cholesterol	19, (36.5)
None	8, (15.4)
Surgical history	
Catheterization	14, (26.9)
Open heart—coronary artery bypass	10, (19.2)
Grafting	3, (5.8)
Open heart—valve replacement	25, (48.1)
None	

Abbreviations: AF = atrial fibrillation, CAD = coronary artery disease, HF = heart failure, HTN = hypertension, kg = kilogram, *n* = number of participants, *N* = total sample size, SD = standard deviation, WHOQOL‐BREF = The World Health Organization Quality of Life‐BREF, Y = years.

**Figure 1:**
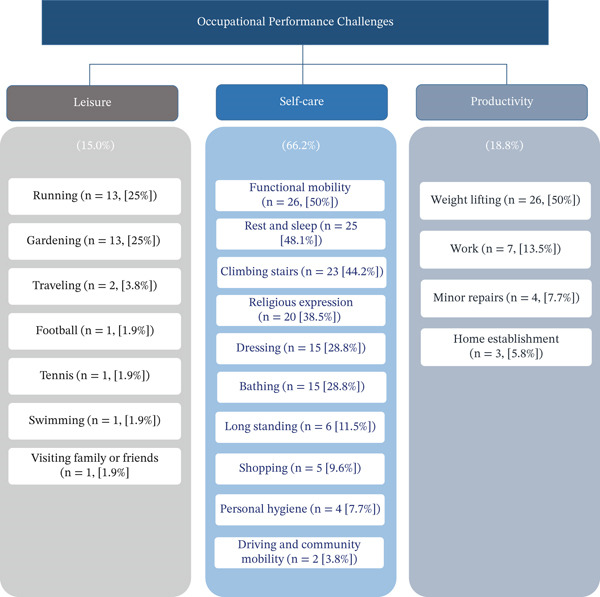
Occupational performance challenges reported by individuals with cardiovascular conditions (*N* = 52). A total of 213 performance challenges were identified. Subcategories within self‐care, productivity and leisure present the performance problem with number and percent of participants who reported each problem.

Descriptive statistics of COPM mean performance and satisfaction scores showed that individuals with cardiovascular conditions perceived their occupational performance (mean = 4.7, SD±1.75, range 1–8) and satisfaction (mean = 4.5, SD±2.04, range 1–10) in the lower moderate range. Participants reported a total of 213 occupational performance challenges within the top five performance problems on the COPM. Self‐care was the most common domain with 141 occupational performance challenges, (66.2% of all reported problems), followed by productivity (18.8%) and leisure (15.0%). Within the self‐care domain, functional mobility was the most frequently reported performance problem as reported by 26 participants (50% of all reported performance problems in self‐care) followed by rest and sleep (*n* = 25 [48.1%]), climbing stairs (*n* = 23 [44.2%]), and religious practices (*n* = 20 [38.5%]). Regarding the productivity domain, lifting heavy objects was the most reported challenging activity (26 participants, 50% of reported problems in productivity), while in the leisure domain, running or gardening were the most commonly reported challenging leisure activities (13 participants, 25% of reported problems in leisure). See Figure 1 for activity examples within the top five reported occupational performance problems.

Descriptive statistics of WHOQOL‐BREF showed that the physical domain mean score was the lowest (M = 50.17, SD±18.4, range = 0–88), followed by the environment (M = 55.33, SD±17.2, range = 13–88), psychological (M = 55.92, SD±16.5, range = 19–94), and social relationships (M = 59.58, SD±23.4, range = 6–100) domains. Around 40 individuals with cardiovascular conditions reported their physical health aspect of QoL as poor, 42% reported their psychological aspect as unstable, 27% reported their social relationships as not active, and around 60% reported their environment as inadequate to achieve a better quality of life (see Table 2).

**Table 2:** COPM and WHOQOL‐BREF scores (*N* = 52).

Scale	Sub‐score classification	Frequencies *n*, (%)	M (±SD), R
COPM	Performance score		4.74 (±1.75), 1–8
	Good (> 5)	18, (34.7)	
	Poor (≤ 5)	34, (65.3)	
	Satisfaction score		4.50 (±2.02), 1–10
	Good (> 5)	15, (28.6)	
	Poor (≤ 5)	37, (71.4)	

WHOQOL‐BREF	Physical domain score		50.27 (±18.21), 5–88
	Good (> 50 points)	26, (50.0)	
	Poor (≤ 50 points)	26, (50.0)	
	Psychosocial domain score		55.92 (±16.54), 19–94
	Stable (> 51 points)	30, (57.7)	
	Unstable (≤ 51 points)	22, (42.3)	
	Social relations domain score		59.58 (±23.44), 6–100
	Active (> 50 points)	34, (65.3)	
	Not active (≤ 50 points)	18, (34.6)	
	Environmental domain score		55.33 (±17.23), 13–88
	Adequate (> 58 points)	22, (42.3)	
	Inadequate (≤ 58 points)	30, (57.7)	

Abbreviations: COPM = Canadian Occupational Performance Measure, M = mean, *n* = number of participants, R = range, SD = standard deviation, WHOQOL‐BREF = World Health Organization Quality of Life Brief version, % = percent.

The physical domain of quality of life was negatively associated with male gender (*r*_*s*_ = −0.37, *p* = 0.007), married social status (*r*_*s*_ = −0.39, *p* = 0.004), high cholesterol (*r*_*s*_ = 0.28, *p* = 0.043), smoking (*r*_*s*_ = −0.35, *p* = 0.009), not practicing sports (*r*_*s*_ = −0.45, *p* = 0.001), and being unemployed (*r*_*s*_ = 0.35, *p* = 0.009). The psychological domain was negatively associated with high cholesterol (*r*_*s*_ = −0.31, *p* = 0.022), while the environment domain was negatively associated with male gender (*r*_*s*_ = 0.38, *p* = 0.005). Mean COPM performance and satisfaction were positively associated with physical quality of life domain (performance: *r*_*s*_ = 0.36, *p* = 0.01; satisfaction: *r*_*s*_ = 0.36, *p* = 0.009), psychological domain (performance: *r*_*s*_ = 0.34, *p* = 0.016; satisfaction: *r*_*s*_ = 0.39, *p* = 0.005), and environment domain (performance: *r*_*s*_ = 0.30, *p* = 0.036; satisfaction: *r*_*s*_ = 0.29, *p* = 0.043) (see Tables 3 and 4).

**Table 3:** Correlation between demographic and condition characteristics, COPM scores, and WHOQOL‐BREF domains (*N* = 52).

	WHOQOL‐BREF domains				COPM scores	
Variables	Physical domain	Psychological domain	Social relations domain	Environment domain	Performance mean score	Satisfaction mean score
Male gender	*r*_s_ = −0.37∗∗ *p* = 0.007			*r*_s_ = −0.38∗∗ *p* = 0.005		
Smoking	*r*_s_ = −0.35∗∗ *p* = 0.009					
Playing sport	*r*_s_ = −0.45∗∗ *p* = 0.001					*r*_s_ = 0.32∗ *p* = 0.022
Single	*r*_s_ = 0.33∗ *p* = 0.014	*r*_s_ = 0.28∗ *p* = 0.043				
Married	*r*_s_ = −0.39∗∗ *p* = 0.004	*r*_s_ = −0.27∗ *p* = 0.045				
Employment status	*r*_s_ = −0.35∗∗ *p* = 0.009				*r*_s_ = 0.29∗ *p* = 0.037	*r*_s_ = 0.33∗ *p* = 0.019
Cholesterol	*r*_s_ = −0.28∗ *p* = 0.043	*r*_s_ = −0.31∗ *p* = 0.022				
Mean COPM performance score (1–10)	*r*_s_ = 0.36∗∗ *p* = 0.010	*r*_s_ = 0.34∗ *p* = 0.016	*r*_s_ = 0.20 *p* = 0.163	*r*_s_ = 0.30∗ *p* = 0.036		*r*_s_ = 0.85∗∗ *p* < 0.001
Mean COPM satisfaction score (1–10)	*r*_s_ = 0.36∗∗ *p* = 0.009	*r*_s_ = 0.39∗∗ *p* = 0.005	*r*_s_ = 0.17 *p* = 0.228	*r*_s_ = 0.29∗ *p* = 0.043	*r*_s_ = 0.85∗∗ *p* < 0.001	

Abbreviations: COPM = Canadian Occupational Performance Measure, WHOQOL‐BREF = World Health Organization Quality of Life Brief version.

∗∗Correlation is significant at the 0.01 level (2‐tailed), *p* < 0.01.

∗Correlation is significant at the 0.05 level (2‐tailed), *p* < 0.05.

**Table 4:** Predictive models of self‐perceived occupational performance and satisfaction with performance (*N* = 52).

Predictor variables	B	95% CI for B [LB–UB]	*β*	SE	*Sig.*	Model fit	
						*R* ^2^	*p*value
a. Predictors of Self‐Perceived Occupational Performance (COPM Performance Mean Scores)							
WHOQOL‐BREF Physical health domain	0.039	[0.012–0.065]	0.397	0.013	0.005∗	0.158	0.005∗
Employed work status	1.109	[0.070–2.148]	0.299	0.516	0.037∗	0.089	0.037∗
b. Predictors of Self‐Perceived Satisfaction with Performance (COPM Satisfaction Mean Scores)							
WHOQOL‐BREF Psychological domain	0.047	[0.011–0.083]	0.350	0.018	0.048∗	0.232	0.048∗
Playing sports	1.138	[0.008–2.268]	0.267	0.561	0.011∗		

Abbreviations: B = unstandardized regression coefficient, CI = confidence interval, COPM = Canadian Occupational Performance Measure, F = F statistic, LB = lower bound, *R* ^2^ = R square value for the predictive model fit, SE = standard error, Sig. = *p* value for each predictor, UB = upper bound, WHOQOL‐BREF = World Health Organization Quality of Life Brief version, *β* = standardized regression coefficient.

∗*p* < 0.05 indicating statistical significance.

When the WHOQOL‐BREF domain scores were added to the regression analysis, a new model emerged in which the physical health domain of QoL was the only significant positive predictor of mean COPM performance, explaining 15.8% of the variance (*R* ^2^ = 0.158, F(2, 47) = 8.810, *p* = 0.005). Significant positive predictors of COPM satisfaction scores included playing sports and having a higher psychological health domain score on the WHOQOL‐BREF, together explaining 23.2% of the variance (*R* ^2^ = 0.232, F(2, 47) = 4.109, *p* = 0.048).

## 5. Discussion and Implications

This study explored occupational performance and satisfaction, and quality of life in individuals with cardiovascular conditions in Jordan. Results revealed that this population perceived their occupational performance in self‐care, productivity, and leisure in the lower moderate range. Functional mobility, sleep, and heavy work were the most challenging daily life activities in this population. Physical and environmental aspects of quality of life were affected. In addition, a number of factors were negatively associated with physical and environmental aspects of quality of life including lower occupational performance and satisfaction in daily life activities, male gender, married social status, and smoking. The following paragraphs discuss these results in details.

It is evident in the literature that individuals with cardiovascular conditions have limited body functions and physical performance skills due to their condition [12, 13]. Our study showed that individuals with cardiovascular conditions perceived their performance and satisfaction in daily life activities as challenging (mean performance and satisfaction were in the lower moderate ranges). Not surprisingly, functional mobility was the most commonly reported challenging activity among participants. Functional mobility involves moving from one position or place to another during performing daily activities, such as in‐bed mobility, wheelchair mobility, functional ambulation, and walking [34]. Many daily life activities require functional mobility to perform them like household activities, going to work, and social activities. Therefore, limited functional mobility significantly impact independent living and quality of life [38]. Result from this current study is aligned with previous research that explored challenging daily life activities in individuals with cardiac diseases, and found that were walking along with some self‐care activities like bathing were challenging [17]. Novel findings in our study—with the use of the COPM—showed additional self‐care challenged activities, like climbing stairs, dressing, and bathing, all of which require functional mobility and exert effort on the cardiovascular system.

It is evident in the literature that cardiovascular conditions affect sleep [39–45]. Chest pain, elevated blood pressure and heart rate, and mechanistic co‐morbid challenges like dyspnea (i.e., shortness of breath) and nocturia (i.e., the need to use the bathroom) might affect sleep quality and duration [43–45]. For example, evidence has showed that individuals with cardiovascular conditions who suffered from nocturia reported significant bother because they needed to visit the bathroom multiple times during the night [44]. Rehabilitation, especially occupational therapy services, ascertain the need for sleep as other daily life activities like eating and learning. Therefore, there is a need to study sleep performance in individuals with cardiovascular conditions in order to support evaluation and intervention services for this population. In this current study, almost half of the participants showed sleep challenges, while did not report having occupational therapy or rehabilitation services. Results from this study ascertain the need for collaboration between medical and rehabilitation personnel to improve function and quality of life in individuals with cardiovascular conditions. While this study showed that sleep was a challenging daily life area, additional studies should investigate more depth sleep as a function in order to support individualized and tailored interventions to promote sleep in individuals with cardiovascular conditions.

Surviving cardiovascular conditions and returning to work and productivity is of tremendous challenge. Many participants in this current study experience heavy duty work as challenging, while some of them also experience light work and minor work duties as problematic. Loss of self‐confidence, anxiety, and physical symptoms might interfere with work duties in individuals with cardiovascular conditions [46]. A systematic review showed common factors across many conditions that might interfere with people’s ability to return to work including older age, being female, higher pain or disability, depression, and higher physical work demands [47]. It is important to highlight here that this current study is showed work and productivity as a challenging daily life area, while previous studies that explored occupational performance in individuals with cardiovascular conditions focused only on self‐care. Comprehensive evaluation of occupational performance using COPM is needed when working with this population in order to understand all challenging daily life areas.

Quality of life is affected in many chronic health conditions [48]. Our results showed that participants with lower occupational performance reported lower physical and environment aspects of quality of life. As discussed earlier, cardiovascular conditions greatly affect individuals’ physical abilities. Knowing that occupational performance refers to engaging in activities in authentic environments of these activities explains the relationship between occupational performance, and physical and environment aspects of quality of life. While there were no previous studies in cardiovascular conditions that highlighted this association, it is worthy to explain here that—besides physical abilities—the environment also contributes to occupational performance and quality of life. Existing studies has showed the role of environmental factors, like life style choices, in causing cardiovascular conditions, e.g., [49, 50]. It is interesting to know from this study that individuals with cardiovascular conditions perceived low quality of life because of limitations in their physical environments. Environmental adaptation, which is an evidence‐based occupational therapy intervention, changes environmental barriers to physical activity and occupational performance in order to promote health, quality of life, and participation [35]. Therefore, the medical intervention for individuals with cardiovascular conditions should include occupational therapy and other rehabilitation services.

Results revealed that the physical health of individuals with cardiovascular conditions predicted better occupational performance in daily life, while their psychological health predicted better occupational satisfaction. Studies that investigated occupational performance and satisfaction across different conditions are scarce; and therefore, the results from this study are worth detailed explanation. Performing many daily life activities like showering or functional mobility require using individuals’ physical abilities like muscle strength and endurance [35]. It is then relevant that limitations in physical abilities due to cardiovascular conditions, e.g., [12] severely restricts individual’s performance in daily life activities. While previous literature has showed that individuals with cardiovascular conditions had limited activity performance, that is, [16–18], the results from this study revealed that the lower physical health of individuals with cardiovascular conditions predicted their poor occupational performance in daily life activities like self‐care, productivity, and leisure. Therefore, in order to improve occupational performance, care providers should focus on improving physical abilities of individuals with cardiovascular conditions, or modifying occupational environments to match individuals’ physical abilities.

There is evidence that show the link between performance satisfaction and wellbeing [51]. Our study has revealed that having better psychological health predicted better performance satisfaction. Also, our results have shown that playing sports and being active predicted better performance satisfaction. A study with similar results but on a different population has shown that psychological health predicted performance and life satisfaction in individuals with neurological conditions [52]. Also, occupational performance and satisfaction were related to physical and psychological health among university students [53]. It is worth to mention here—because there is scare related literature about it—the importance of exercise in improving physical health, and therefore, occupational performance and satisfaction. Cardiorespiratory fitness was related to life satisfaction in adults [54]. Also, participation in meaningful everyday life activities in individuals with stroke was linked to life satisfaction [55]. Therefore, our results suggest the significance of designing therapeutic interventions to improve individuals’ physical and psychological abilities of individuals with cardiovascular conditions.

### 5.1. Study Strengths, Limitations, and Future Directions

Unlike previous studies in this field, this study utilized the COPM in order to show challenging activities in all daily life areas in individuals with cardiovascular conditions. Also, this study included individuals with stable cardiac conditions, whereas previous studies mainly focused on heart failure. While the COPM captures individualized abilities and challenges, it is still a subjective measure of occupational performance. Using another objective measure of performance, like the Functional Performance Measure, might be more rigorous and comprehensive. Also, using a complementary movement tool like the accelerometer might enhance understanding motor challenges in individuals with cardiovascular conditions. Although it was challenging to recruit 52 participants with cardiovascular conditions, the small sample size represents an additional limitation of this study particularly for conducting a regression analysis with multiple predictors. Recruitment via social media platforms is considered easy and efficient compared to hospital settings, yet they may raise biased samples (e.g., selection and unequal access bias) because the proportion of the whole population remains unknown [56]. Also, participants in this study might be from higher socioeconomic backgrounds or are technology users because they had exposure to study invitation via social media platforms. This may explain the younger age profile and male predominance of the sample, which justifies the need for future research targeting older adults through in‐person recruitment and data collection methods.

While phone interviews were efficient in saving time, effort, and transportation for participants, they might increase the risk of information bias due to the lack the face‐to‐face rapport building and the inability to as assess nonverbal communication cues. Similar to other cross‐sectional studies, this study was quick to study and analyze multiple variables that were related to occupational performance in individuals with vascular conditions. However, the cause and effect relationship cannot be derived from the current study. As for future research should investigate occupational performance with more sample size, and more rigorous designs. Intervention and longitudinal studies are needed to investigate the effect of personal and environmental factors on occupational performance in this population. Furthermore, future studies should focus on the objective assessment of physical functions among individuals with cardiovascular conditions to determine their impact on occupational performance and satisfaction with performance.

Future research should thoroughly explore personal factors (i.e., educational level, body mass index, daily diet, and exercise routine) and environmental factors (house arrangement and accessibility) on occupational performance and quality of life in individuals with cardiovascular conditions. Additionally, future research should thoroughly investigate the effectiveness of environmental adaptation interventions in enhancing occupational performance and quality of life in individuals with cardiovascular conditions. For example, intervention studies to investigate the effect of home environment modification on improving functional mobility in individuals with cardiovascular conditions are highly needed. Also, investigating the effect of adapting the work environment on return to work in this population is under investigated and worth research expansion.

## 6. Conclusion

This study revealed that individuals with cardiovascular conditions perceived their occupational performance and satisfaction in self‐care, productivity, and leisure in the lower moderate range. In addition, functional mobility, sleep, and heavy work were the most challenging daily life activities that affected aspects of quality of life in this population. Interventions to support occupational performance in individuals with cardiovascular conditions should target individualized and tailored interventions to improve functional mobility, sleep, and work activities. Health care providers should consider enhancing physical abilities, as well as, environmental supports in order to improve quality of life in this population. For example, home environment adaptations (e.g., moving bedroom to down stairs) enhance functional mobility, and tailored diet and exercise routines improve physical abilities in individuals with cardiovascular conditions.

## Author Contributions

A.J. and R.A. researched literature and conceived the study. All authors were involved in protocol development. A.J., R.A., and S.R. were involved in gaining ethical approval and patient recruitment. A.J., M.N., A.O., and S.N. were involved in the data analysis, reporting of results, and development of the discussion section. A.J., N.I., R.A., and M.N. wrote the first draft of the manuscript.

## Funding

This study was supported by Jordan University of Science and Technology, 20200205; Erasmus+, 573758‐EPP‐1‐2016‐1‐JO‐EPPKA2‐CBHE‐JP.

## Disclosure

All authors reviewed and edited the manuscript and approved the final version of the manuscript.

## Ethics Statement

Institutional Review Board of Jordan University of Science and Technology approved the study (Research ID # 20200205, IRB#:19/131/2020, approval date: March 8, 2020).

## Consent

Verbal informed consent to participate was obtained from all participants. The verbal informed consent also included consent for publication. Written informed consent was not applicable because the study was conducted via phone interviews.

## Conflicts of Interest

The authors declare no conflicts of interest.

## Data Availability

Data available on request due to privacy/ethical restrictions.
